# Ubiquitination-Dependent Cyclin Regulation in Gastric Cancer and Emerging Targeted Therapies with Chinese Herbal Bioactive Compounds

**DOI:** 10.3390/cancers18193179

**Published:** 2026-10-02

**Authors:** Sicheng Qian, Xinyi Zheng, Jiaqi Li, Jiahan Le, Jiangjiang Qin, Yitao Chen

**Affiliations:** 1 School of Life Sciences, Zhejiang Chinese Medical University, Hangzhou 310053, China; 202311115511051@zcmu.edu.cn (S.Q.);; 2 Jinhua Academy, Zhejiang Chinese Medical University, Jinhua 321000, China; 3 Hangzhou Institute of Medicine (HIM), Chinese Academy of Sciences, No. 150 Dongfang Street, Qiantang District, Hangzhou 310053, China

**Keywords:** ubiquitin, gastric cancer, cell cycle, cyclins, E3 ubiquitin ligase, traditional Chinese medicine

## Abstract

Gastric cancer (GC) remains one of the most prevalent malignancies worldwide, with a high mortality rate primarily attributable to late-stage diagnosis and limited therapeutic options. Aberrant cell proliferation arising from cell cycle dysregulation is a hallmark of GC pathogenesis. Cyclins, as core regulators of cell cycle progression, exhibit dynamic protein expression patterns that are tightly controlled by the ubiquitin–proteasome system (UPS). In GC, cyclins are frequently overexpressed due to impaired ubiquitination and subsequent proteasomal degradation. This review systematically elucidates the ubiquitination-mediated regulation of distinct cyclins (Cyclins D, E, A, and B) at specific cell cycle phases—G1, S, G2, and M—and highlights the critical roles of E3 ubiquitin ligases, including SCF complexes and APC/C, in GC progression. We further discuss how dysregulated non-coding RNAs and altered E3 ligase expression contribute to aberrant cyclin ubiquitination, thereby promoting GC cell proliferation, metastasis, and poor prognosis. Importantly, emerging evidence demonstrates that bioactive compounds derived from traditional Chinese medicine (TCM) can counteract these abnormalities by directly downregulating cyclin expression, modulating E3 ligase activity, or inhibiting 26S/20S proteasome function, thus restoring cell cycle homeostasis and suppressing GC growth. This review integrates molecular mechanisms of cyclin ubiquitination with TCM-based therapeutic strategies, offering novel insights into targeted interventions for GC.

## Simple Summary

Gastric cancer remains difficult to treat, and uncontrolled cell division is a major reason why it grows and spreads. Proteins called cyclins act as timers that move cells through division, while a cellular disposal system removes them after use. In gastric cancer, this removal process is often disrupted, allowing cyclins to build up and drive tumor growth, spread, and treatment resistance. This review explains how cyclins are marked and destroyed at each stage of cell division and identifies the enzymes and regulatory molecules that disturb this process. It also summarizes evidence that bioactive compounds from traditional Chinese medicine may restore cell cycle control by lowering cyclin levels, altering the enzymes that mark proteins for destruction, or affecting the protein-disposal machinery. These insights may reveal new treatment targets and guide development of combination therapies.

## 1. Introduction

Gastric cancer (GC) is the fifth most commonly diagnosed cancer and the fourth leading cause of cancer-related deaths globally, with over one million new cases annually and a five-year survival rate of only 20% for advanced stages due to late detection [1,2]. Although early screening and endoscopic resection have reduced mortality in some regions, aging populations and persistent risk factors—such as Helicobacter pylori infection, a high-salt diet, smoking, and alcohol consumption—contribute to an increasing GC burden [3,4,5,6,7,8]. Current treatments for advanced GC include chemotherapy, immunotherapy (e.g., anti-PD-1/CTLA-4 agents), and targeted therapies, yet prognosis remains dismal, underscoring the urgent need for novel therapeutic approaches [9,10,11].

At the molecular level, uncontrolled cell proliferation driven by cell cycle dysregulation is a key event in GC initiation and progression [12,13,14,15]. Cyclins, which activate cyclin-dependent kinases (CDKs) to govern cell cycle transitions, are essential for proper mitotic progression. Their protein levels fluctuate tightly during the cell cycle, and their timely degradation is executed by the ubiquitin–proteasome system (UPS). Ubiquitination, a post-translational modification mediated by the sequential action of E1 (activating), E2 (conjugating), and E3 (ligase) enzymes, tags cyclins with polyubiquitin chains (notably K48- and K11-linked) for proteasomal destruction [16,17,18,19,20]. Among the E3 ligases, the Skp1-Cullin-F-box (SCF) complex primarily governs Cyclins D, E, A, and F ubiquitination from the G1 to the G2 phase, whereas the anaphase-promoting complex/cyclosome (APC/C) controls Cyclin B degradation during mitosis [18,19,20]. Disruption of this ubiquitination machinery leads to cyclin accumulation, aberrant cell cycle progression, and tumorigenesis [21].

In GC, numerous studies have documented abnormal cyclin overexpression resulting from defective ubiquitination. This dysregulation is often attributed to altered expression or function of E3 ligases (e.g., FBXW7, FBXO4, SKP2, AMBRA1, TRIM family members) and regulatory non-coding RNAs (miRNAs, lncRNAs, circRNAs) that modulate ubiquitination efficiency. Moreover, recent research has revealed that active ingredients from traditional Chinese medicine (TCM) can interfere with cyclin ubiquitination either by directly suppressing cyclin expression or by targeting E3 ligases and proteasome activity, thereby restoring cell cycle control and inhibiting GC progression.

In this review, we provide a comprehensive and up-to-date overview of ubiquitination-dependent cyclin regulation at each cell cycle phase in GC, detailing the specific E3 ligases, E2 enzymes, and accessory factors involved. We further summarize the anti-GC mechanisms of TCM bioactive compounds that act on the UPS–cyclin axis, with the aim of identifying potential therapeutic targets and guiding future drug development for this devastating disease.

**Figure 1:**
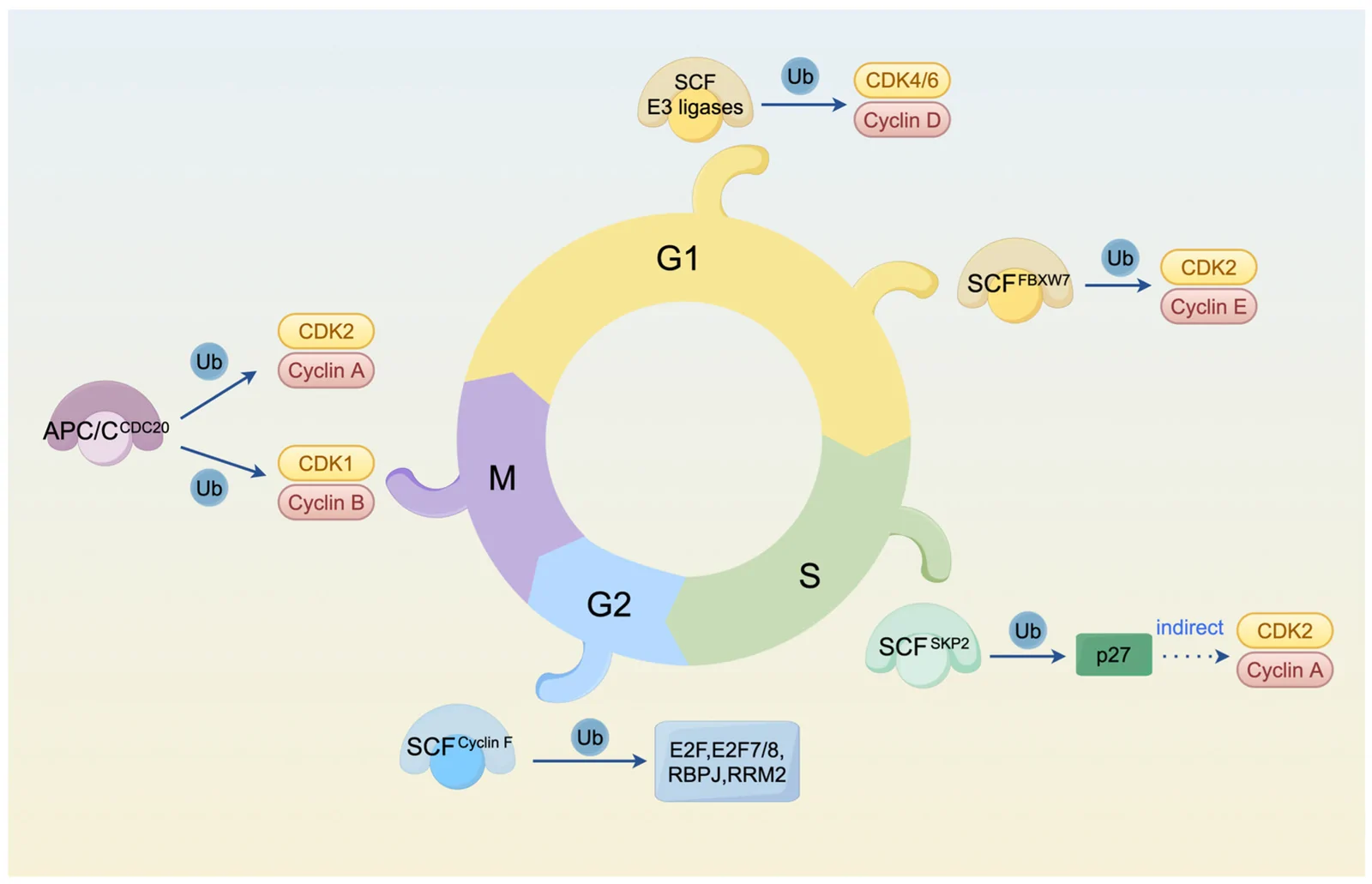
Direct and indirect ubiquitination-related regulation of cyclins across the cell cycle. Solid arrows indicate direct ubiquitination of the indicated substrates, whereas the dashed arrow indicates indirect activation of Cyclin A-CDK2 following SCF^SKP2^-mediated degradation of p27. SCF^Cyclin F^ ubiquitinates non-cyclin substrates. During mitosis, APC/C^CDC20^ promotes Cyclin A degradation before Cyclin B is degraded.

The mechanisms discussed in this review fall into three groups: direct ubiquitination of cyclins; indirect regulation of cyclins through ubiquitination of non-cyclin substrates; and SCF^Cyclin F^-mediated ubiquitination, in which Cyclin F serves as the F-box substrate-recognition subunit rather than as the ubiquitinated substrate (Figure 1).

## 2. Ubiquitination of Cyclins in the G1 Phase of GC

The G1 phase of the cell division cycle is a critical period for cell proliferation, during which chromatin is replicated, opening a window of opportunity for determining cell fate [22]. In this period, cyclins, specifically Cyclin D and Cyclin E, and the ubiquitination-mediated degradation of cyclins play a crucial role. The degradation of cyclins mediated by the ubiquitin–proteasome system drives the progression of the cell cycle to the next phase. In cancer development, the ubiquitination-mediated degradation of related proteins becomes abnormal, disrupting the normal progression of the cell cycle [23]. Cyclin-dependent kinase 4/6 (CDK4/6) is activated by Cyclin D in the early G1 phase and cyclin-dependent kinase 2 (CDK2) is activated by Cyclin E in the late phase to form an active complex. Then Cyclin D-CDK4/6 and Cyclin E-CDK2 phosphorylate pRB, ultimately leading to dissociation from E2F, thereby promoting the transition of the cell cycle from the G1 phase to the S phase [24,25]. However, in cancer occurrence, alterations in the ubiquitination of relevant proteins can lead to disruptions in the normal progression of the cell cycle.

### 2.1. The Ubiquitination of Cyclin D

Cyclin D is a highly unstable protein, with its degradation mainly occurring in the late G1 and early S phases. Under physiological conditions from the G1 to the S phase, glycogen synthase kinase-3β (GSK-3β) phosphorylates Cyclin D1 at Thr-286, prompting its relocation from the nucleus to the cytoplasm. Subsequently, Cyclin D1 is targeted for ubiquitination by the SCF ubiquitin ligase and degraded by the 26S proteasome [26]. Abnormal expression of Cyclin D is one of the main causes of GC, as its dysregulated ubiquitination and degradation not only lead to cancer but also diminish the therapeutic efficacy of CDK4/6 inhibitors.

**Figure 2:**
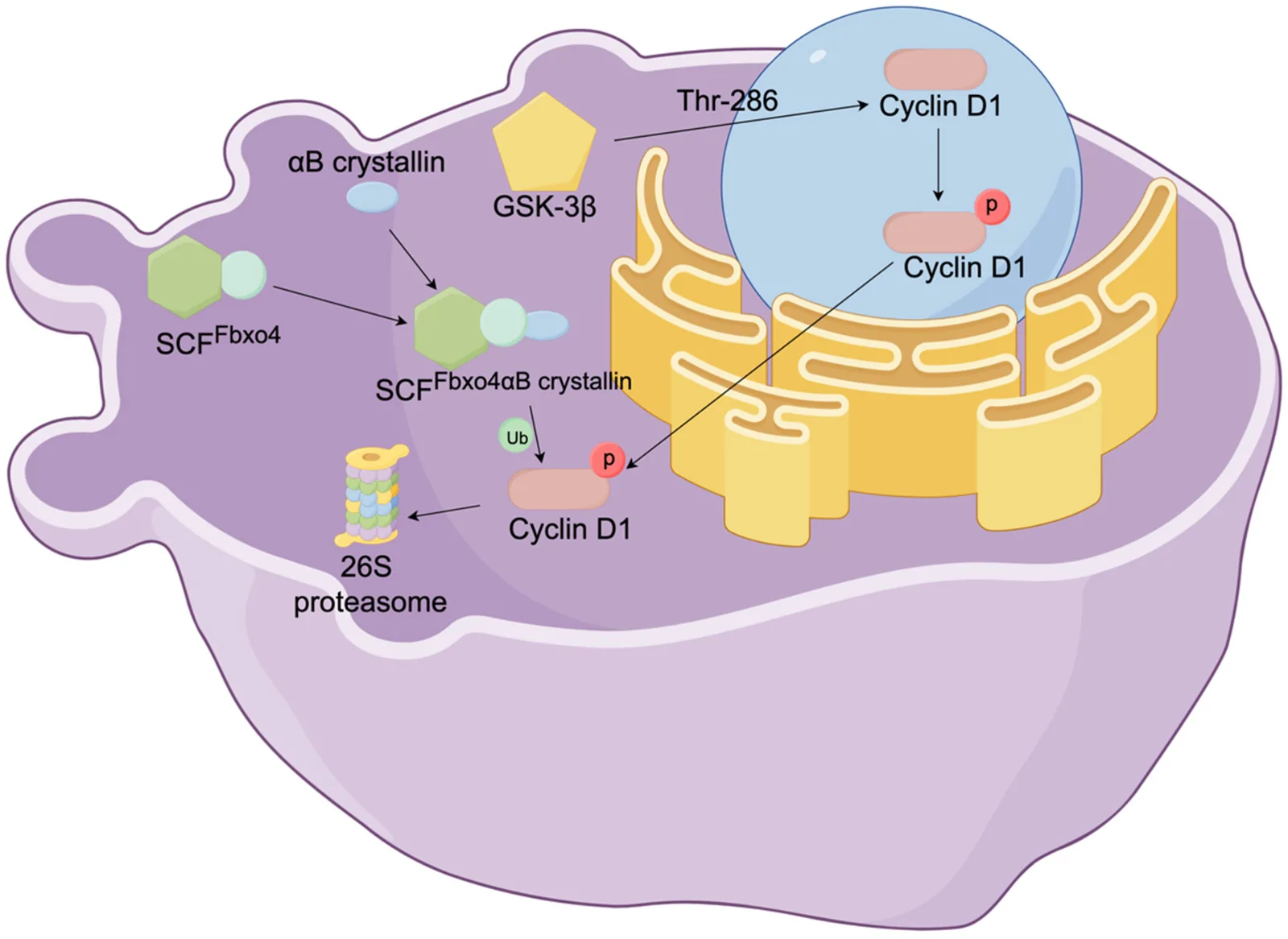
SCF^Fbxo4-αB crystallin^ mediates the ubiquitination of Cyclin D1 and promotes its degradation by the 26s proteasome.

Research indicates that Fbxo4 and Fbxo31 can regulate the ubiquitination degradation of Cyclin D1 to maintain proper cell cycle progression. Fbxo4 binds with αB crystallin to form SCF^Fbxo4-αB crystallin^. This complex accepts Ub, which is activated by UBA1 and UBA6 and transferred by the UBE2D family, and rapidly ubiquitinates Cyclin D1, directing it for degradation by the 26S proteasome (Figure 2) [27]. In tumorigenesis, reductions in αB crystallin and Fbxo4 hinder the ubiquitination of Cyclin D1 and elevate cytoplasmic hnRNPK levels, thereby augmenting its interaction with c-Myc and facilitating the translation and accumulation of c-Myc protein, which can promote the transcription of Cyclin D1, ultimately fostering aberrant cell proliferation and metastasis [28,29]. On the other hand, Fbxo31 mediates the ubiquitination of Cyclin D1 by binding to its C-terminal FBXO31 β-barrel with the free C-terminal carboxylate tail of Cyclin D1 [30]. Research indicates that elevated levels of miR-17 and miR-20a in GC significantly decrease Fbxo31 mRNA and protein expression, inhibiting the degradation of Cyclin D1 and causing dysregulation of the cell cycle [31].

In conjunction with Fbxo4 and Fbxo31, the E3 ubiquitin ligase CRL4^AMBRA1^ exhibits the ability to ubiquitinate Cyclin D, encompassing all isoforms of Cyclin D (Cyclin D1, Cyclin D2, and Cyclin D3), and facilitate its degradation through the proteasome. After UBA1 is bound to Ubs, E2 ubiquitin-conjugating enzymes such as UBE2D1, UBE2G1, and UBE2G2 associated with the CUL4 complex transfer Ub to CRL4^AMBRA1^. This results in the attachment of Ub to Cyclin D, leading to its degradation by the 26S proteasome. This enables the seamless transition of cells from the G1 phase to the S phase, ensuring genomic integrity during DNA replication and thereby modulating the advancement of the cell cycle [32,33]. Prior to this, AMBRA1 has been found to be associated with neurodevelopmental defects related to cell proliferation and apoptosis, while also being an efficient tumor suppressor gene [34,35]. Abnormal expression of AMBRA1 may lead to aberrant degradation of Cyclin D, thereby affecting the progression of the cell cycle and leading to the occurrence of GC. Research conducted by Qu et al. has demonstrated that the expression of AMBRA1 in GC cells is notably higher compared to normal cells, and this elevated expression is significantly correlated with the progression of GC, indicating a worse prognosis [36]. The increased expression of MG53 (also known as TRIM72) can impair insulin signaling and glucose metabolism, triggering muscle insulin resistance and metabolic syndrome [37]. However, as an E3 ligase, some studies have shown that MG53 can also function as a tumor suppressor. MG53, along with other E3 ubiquitin ligases of the TRIM family, can accept Ubs transferred from UBA1, the UBE2D family, and UBE2N, and ubiquitinate Cyclin D1 via K48 linkage. Fang et al. found that the expression of MG53 significantly decreases in GC and is positively correlated with patient survival rates. The decrease in MG53 expression inhibits the ubiquitination of Cyclin D1, resulting in reduced ubiquitin-mediated degradation. The excess Cyclin D1 leads to GC cells being unable to smoothly transition from the G1 to the S phase, causing arrest in the G1 phase [38].

Additionally, a study suggests that the proliferation and migration of GC cells are regulated by the hsa_circ_001988/miR-197-3p/FBXW7 axis [39]. In GC cells, decreased expression of hsa_circ_001988 attenuates the inhibition of miR-197-3p expression, subsequently leading to decreased expression of FBXW7. FBXW7, functioning as an E3 ligase, plays a crucial role in modulating protein expression, such as Cyclin D1, thereby impacting cell cycle regulation. When the expression of FBXW7 in GC cells is downregulated due to abnormal expression of hsa_circ_001988, it leads to an increase in Cyclin D1 protein levels, ultimately resulting in the disruption of the cell cycle and abnormal proliferation of tumor cells.

Skp2 can influence cell proliferation by promoting the ubiquitination degradation of Cyclin-dependent kinase inhibitors (CKIs). CKIs are classified into two categories based on their structure and binding with CDKs: the inhibitors of the Cdk4 (INK4) family, including p16^INK4a^, p15^INK4b^, p18I^NK4c^, and p19^INK4d^ [25,40,41,42,43,44], and the CIP/KIP family, including p21^Cip1^, p27^KIP1^, and p57^KIP2^ [44,45,46]. The INK4 family specifically inhibits the catalytic subunits of CDK4 and CDK6, blocking their binding to Cyclin D and promoting the degradation of Cyclin D. The CIP/KIP family, on the other hand, inhibits p21 and p27, leading to a decrease in the stability of Cyclin D, as p21 and p27 can enhance Cyclin D stability [47]. When GC cells enter the G1 phase, the increased levels of Skp2 lead to enhanced ubiquitination of both the INK4 family and the CIP/KIP family, promoting their degradation by the proteasome, thereby promoting the stability of Cyclin D and resulting in abnormal accumulation of Cyclin D [48]. Studies have also found that in GC, SKP2 levels are influenced by mTORC1. mTORC1 can bind to Skp2 and inhibit its ubiquitination degradation by phosphorylating Ser64, resulting in Skp2 overexpression, thereby affecting the cell cycle [49].

Another study indicated that in GC, the mRNA and protein levels of tripartite motif 59 (TRIM59) are significantly increased. TRIM59 promotes tumor development by interacting with p53 to promote its ubiquitination and degradation, thereby inhibiting downstream signaling of p53. Interestingly, Zhou et al. found that TRIM59 expression is also positively correlated with Cyclin D1 [50]. In the study by Pan et al., the overexpression of Ubiquitin-conjugating enzyme E2S (UBE2S) promoted the ubiquitination of p53, which in turn affected the expression of downstream targets p21 and Cyclin D1, thereby enhancing cell proliferation and migration [51]. Meanwhile, tripartite motif 58 (TRIM58) can regulate the progression of GC by mediating the ubiquitination degradation of β-catenin. As a tumor suppressor protein, TRIM58 expression is typically downregulated in GC, further leading to upregulation of β-catenin and Cyclin D1 expression, promoting the proliferation of GC cells [52]. Furthermore, Lin et al. found that ubiquitin-associated protein 2-like (UBAP2L) is also highly expressed in GC, activating the Wnt/β-catenin signaling pathway, thereby promoting the accumulation of nuclear β-catenin and the expression of downstream targets including Cyclin D1 [53].

The RNA-binding protein Insulin-like growth factor II mRNA binding protein 3 (IGF2BP3) enhances the stability of Cyclin D1 mRNA, and its expression levels are positively correlated with the incidence of various cancers, including gastric cancer. circNFATC3, a circRNA that can bind to IGF2BP3, has also been detected to be upregulated in GC [54]. Studies have shown that circNFATC3 can inhibit the ubiquitination of IGF2BP3 mediated by TRIM25 by binding to IGF2BP3. In GC, overexpression of circNFATC3 reduces the ubiquitination degradation of IGF2BP3, thereby increasing Cyclin D1 expression and promoting the proliferation and migration of GC cells.

RN181, also known as RNF181 or HSPC238, is a RING finger domain-containing protein that also functions as an E3 ubiquitin ligase [55]. During the G1/S transition phase, RN181 can downregulate the activity of Cyclin D1-CDK4 by inhibiting the ERK/MAPK signaling pathway through ubiquitination to ensure the smooth transition of the cell cycle from the G1 to the S phase. When GC occurs, the expression of RN181 is significantly reduced, leading to an increase in the activity of Cyclin D1-CDK4, which promotes the proliferation of GC cells.

### 2.2. The Ubiquitination of Cyclin E

Cyclin E can bind and activate Cdk2, catalyzing the transition of the cell cycle from the G1 phase to the S phase [56]. Aberrant expression of Cyclin E is observed in many cancers, including GC [57], and gene amplification and abnormal expression of Cyclin E play crucial roles in the aberrant growth and development of GC. In GC, tumors with high Cyclin E expression exhibit higher invasiveness and have a poorer prognosis compared to those with low expression. Excessive Cyclin E inhibits the APC/C^Cdh1^ complex during mitosis, leading to chromosomal misalignment and polyploidy, thereby affecting cell cycle regulation [58]. Human epidermal growth factor receptor 2 (HER2), an oncogene, is frequently overexpressed in GC [59]. And in GC cells with high HER2 expression, Cyclin E expression levels also increase, and patients with tumors showing high Cyclin E expression have worse survival rates and are associated with resistance to trastuzumab [60,61].

The ubiquitin-mediated proteasomal degradation of Cyclin E primarily occurs through two pathways. One involves the degradation of unphosphorylated Cyclin E monomers via the BTB-Cul-3-Rbx1 (BCR) pathway [62], and the other involves the ubiquitination degradation of Cyclin E-Cdk2 mediated by SCF^FBXW7^ [56,63]. FBXW7 transfers Ub from UBE2R1 (also known as CDC34) to Cyclin E-Cdk2, via K48 linkage, mediating the ubiquitination and degradation of Cyclin E-Cdk2. In physiological conditions, phosphorylation of residues Thr395, Ser399, and Thr77 triggers the ubiquitination of Cyclin E, facilitated by phosphorylation at the N- and C-terminals via GSK3 kinase mediation, thereby enhancing recognition by FBXW7 [64,65]. In tumor cells, inactivation of p53 leads to decreased expression of FBXW7, inhibiting its ubiquitination degradation of Cyclin E, resulting in spindle dysfunction and upregulation of c-Myc expression, further impacting the cell cycle [66,67,68]. Studies have shown that heterozygous deletion of FBXW7 is detected in over 30% of early-onset gastric cancers (EOGCs), and decreased FBXW7 expression is closely associated with poor prognosis [67,69,70]. Additionally, Tan et al. found that PP2A-B55β can dephosphorylate Cyclin E, protecting it from ubiquitination by SCF^FBXW7^. In cancer, the aberrant overexpression of B55β leads to the dephosphorylation of Cyclin E, rendering it unable to be properly ubiquitinated by SCF^FBXW7^ and degraded by the proteasome. This results in increased accumulation of Cyclin E, thereby promoting abnormal proliferation of cancer cells [65].

In addition to the direct ubiquitination degradation targeting Cyclin E, aberrant ubiquitination degradation of proteins related to Cyclin E can indirectly affect Cyclin E expression and ultimately impact cell proliferation and cell cycle progression. In GC cases, the expression of Cyclin E is negatively correlated with p21 [71], while the expression of long non-coding RNAs (lncRNAs) is often dysregulated and controls tumor development and progression. Hepatocyte nuclear factor 1 homeobox A antisense RNA 1 (HNF1A-AS1) is an lncRNA and significantly upregulated in GC, and its overexpression enhances cell proliferation and cell cycle progression [72]. The binding of HNF1A-AS1 to miR-661 promotes the expression of CDC34, thereby further increasing the ubiquitination of p21 mediated by CDC34, ultimately leading to an elevation in the levels of Cyclin E. Additionally, overexpression of HNF1A-AS1 and Early Growth Response Protein 1 (EGR1) can enhance the expression of Cyclin E1 as well as CDK2 and CDK4, further increasing the accumulation of Cyclin E, leading to cell cycle abnormalities. Niu et al. proved Calcyclin binding protein/Siah-1 interacting protein (CacyBP/SIP) undergoes nuclear translocation under gastric protein induction, promoting the development of GC cells in the G1 phase [73]. They discovered that following nuclear translocation, CacyBP/SIP interacts with SKP1 to facilitate the ubiquitination of the cell cycle inhibitor p27^Kip1^ by SCF^SKP1^, leading to elevated Cyclin E protein levels and ultimately stimulating the proliferation of GC cells.

miRNAs also play crucial roles in tumor development, and various miRNAs in GC have been identified as biomarkers and prognostic factors [74,75,76]. Studies have shown that miR-25 can suppress AGS apoptosis in GC cell lines by regulating the expression of Cyclin E and MYC [77]. They found that in GC, the expression profile of miR-25 is significantly upregulated, thereby inhibiting the expression of FBXW7. Cyclin E1, as a key downstream target protein of FBXW7, can be ubiquitinated by FBXW7. In the absence of FBXW7 regulation, Cyclin E expression level is indirectly increased by miR-25, thereby enhancing the viability of AGS and inhibiting AGS apoptosis (Table 1).

## 3. Ubiquitination of Cyclin A in the S Phase of GC

After the G1 phase, the S phase follows promptly. The cyclin that plays a primary role in the S phase is Cyclin A, which appears during the G1/S transition and gradually accumulates in the S phase and rapidly disappears after mitosis. Cyclin A plays a crucial role in DNA replication and the initiation of mitosis throughout the cell cycle [78,79]. It can activate two different CDKs, CDK1 and CDK2. The activation of CDK1 and CDK2 requires binding to a cyclin subunit and phosphorylation on a threonine residue on a loop structure (T-loop) located near the mouth of the active site. Specifically, CDK1′s phosphorylation site is Thr161, while CDK2′s phosphorylation site is Thr160 [78]. The ubiquitin-specific protease USP37 can bind specifically to Cdh1 and is related to Cyclin A [80]. In the late G1 phase, Cyclin E-CDK2 phosphorylates USP37, activating its deubiquitination function and preventing its ubiquitination by APC/C^Cdh1^. The APC/C^Cdh1^-USP37 complex avoids the ubiquitination of Cyclin A, promoting Cyclin A accumulation. Cyclin A-CDK2 further activates USP37, forming a positive feedback loop that allows cells to enter the S phase smoothly [81].

Cyclin A-Cdk2, like Cyclin B1-Cdk1 and Cyclin E-Cdk2, can phosphorylate p27 at Thr187, allowing it to be ubiquitinated by SCF^SKP2^ and finally degraded by the proteasome. And Cyclin A-Cdk2, by enhancing SCF^SKP2^ ubiquitination activity in a non-catalytic form, has a better ubiquitination-promoting effect [82,83]. But only stable Cyclin A-Cdk2 can phosphorylate p27. Additionally, Cyclin A is negatively regulated by p27, which can inhibit CDKs and cyclins, ultimately limiting the rate of cell cycle transition from the G1 phase to the S phase, allowing the cell cycle to proceed smoothly [84,85]. Furthermore, studies have shown that SKP2 expression levels are high in the S phase and its association with Cyclin A is crucial for cell cycle regulation. The interaction between SKP2 and Cyclin A protects Cyclin A-Cdk2 from inhibition by p27. Meanwhile, SKP2 can ubiquitinate CKIs with the assistance of E1 ubiquitin-activating enzymes, like UBA1, and E2 ubiquitin-conjugating enzymes such as the UBE2D family, leading to their degradation. The degradation of CKIs activates the activity of Cyclin A-Cdk2, Cyclin A-Cdk2 phosphorylates Cdh1, and Cdh1 is ultimately dissociated from APC/C [86]. When GC occurs, it often accompanies an increase in the expression of SKP2, resulting the accumulation of Cyclin A. By disrupting the interaction between SKP2 and Cyclin A, selective killing of tumor cells can be achieved, effectively suppressing cancer [87].

## 4. Cyclin A Function and SCF^Cyclin F^-Mediated Substrate Ubiquitination in the G2 Phase of GC

During G2, Cyclin A continues to accumulate and promotes mitotic entry, whereas Cyclin F reaches peak abundance [88]. Because APC/C-dependent degradation of Cyclin A begins in prometaphase, after mitotic entry, it is discussed together with Cyclin B degradation in Section 5 [89].

### 4.1. Cyclin A Function and Indirect Ubiquitination-Related Regulation During G2

Cyclin A is crucial for initiating mitosis normally, Fung et al.’s research indicates that the absence of Cyclin A can cause cells to arrest in the G2 phase [90]. Furthermore, Hégarat et al. using PCNA-mRuby fusion, demonstrated that Cyclin A needs to be present or be newly synthesized in the G2 phase before entering the M phase [91]. Regarding how Cyclin A promotes cells from G2 into mitosis, studies have found that Cyclin A-CDK1 phosphorylates and activates Bora. Activated Bora, in conjunction with Aurora A, phosphorylates and activates Plx1, which further activates a positive feedback loop of Cyclin B-CDK1, promoting cell entry into mitosis [92].

Like other cancers, resistance to DNA damage is a hallmark of GC [93]. At the G2/M checkpoint, when DNA damage occurs, CDK2 plays a crucial role. ATM phosphorylates checkpoint kinase Chk2, which in turn phosphorylates p53, activating the apoptosis pathway. At this time, certain deubiquitinases (DUBs), such as USP7 and USP8, play critical roles. USP28 stabilizes Chk2 at the DNA damage checkpoint and regulates the Chk2-p53-PUMA pathway, promoting apoptosis [94,95,96].

Ring finger protein 6 (RNF6) is a member of the E3 ubiquitin-ligase family. It primarily regulates the function of target proteins by forming K48 and K63 Ub chains in collaboration with E1 enzymes such as UBA1 and E2 enzymes like the UBE2D family. K48 Ub chains are mainly associated with protein degradation. RNF6 also plays a role in DNA binding and transcriptional regulation and is involved in important pathways such as tumor cell cycle and apoptosis. Studies have demonstrated that downregulation or upregulation of RNF6 expression can inhibit or promote the growth of GC cells [97,98]. Cyclin A and CREB binding protein (CREBBP) are downstream targets of RNF6 transcriptional regulation in GC. RNF6 upregulates their transcription in GC cells by binding to the promoter of Cyclin A/CREBBP, promoting GC development [98].

Evdokimova et al. found that the upregulation of Y-box binding protein 1 (Ybx1) expression has been associated with cancer invasiveness and can promote cell proliferation by increasing the transcription of Cyclin A and Cyclin B1 [99]. The E3 ubiquitin ligase Nedd4l can ubiquitinate substrate proteins with the assistance of UBA1, UBE2L3, and UBE2D1. Studies have found that Nedd4l degrades Ybx1 through the Ub–proteasome pathway [100]. In cancer, the upregulation of Ybx1 expression increases the transcription of Cyclin A, promoting its accumulation and resulting in cell cycle abnormalities. Evidence that NEDD4L ubiquitinates YBX1 was obtained in a cardiac model rather than a GC model, and the relevance of this mechanism to GC has not yet been established.

### 4.2. SCF^Cyclin F^-Mediated Ubiquitination of Non-Cyclin Substrates

Cyclin F was initially identified as a cyclin-related protein, but unlike canonical cyclins, it does not activate a CDK [101]. Instead, it functions as the F-box substrate-recognition subunit of the SCF^Cyclin F^ E3 ligase [101,102,103]. This subsection therefore concerns ubiquitination mediated by Cyclin F rather than ubiquitination of Cyclin F itself. Evidence for the non-cyclin substrates discussed below, including E2F1, E2F7/E2F8, RBPJ, and RRM2, comes mainly from non-GC models, and their roles in GC remain to be determined.

Members of the E2F family are classified as either transcriptional activators or repressors. E2F1, E2F2, and E2F3a are known as mature transcriptional activators, promoting the transition from the G1 phase to the S phase by controlling Cyclin E and Cyclin A [24,25]. On the other hand, E2F7 and E2F8 are transcriptional repressors classified as “atypical”, and their expression is associated with high cell proliferation rates [104,105]. During the transition from the S phase to the G2 phase, SCF^Cyclin F^ can ubiquitinate E2F1 with the assistance of UBA1 and UBE2R1, leading to its degradation by the 26S proteasome. Loss of Cyclin F leads to increased levels of E2F1 in the G2 phase, particularly detrimental for inhibiting DNA damage checkpoint kinase Chk1 [106]. Additionally, Cyclin F can ubiquitinate E2F7 and E2F8 and causes them to be degraded by the proteasome in the G2 phase. Studies have shown that only when E2F7 and E2F8 are degraded can cells smoothly transition through the G2/M phase [107,108]. Using CRISPR-CAS9 technology [109], Yuan et al. validated that knocking down Cyclin F rescued the delay in cell cycle progression caused by the knockdown of E2F7 and E2F8, while upregulation of E2F7 and E2F8 expression resulting from Cyclin F knockdown led to the failure of the G2/M phase transition [107].

Isocitrate dehydrogenase 1 (IDH1) is a key enzyme catalyzing the interconversion of isocitrate and 2-oxoglutarate (2-OG) and is found to be mutated in various cancers, with the most common mutations being caused by the R132 mutation [110,111]. The loss of Cyclin F contributes to IDH1^R132H^-mediated tumorigenesis and metastasis. Additionally, IDH1 has been identified as a transcriptional target of Recombination signal binding protein for immunoglobulin kappa J region (RBPJ). Metabolic stress-induced Cyclin F induces the ubiquitination of RBPJ at Lys315, leading to its proteasomal degradation. Thus, Cyclin F can inhibit IDH1^R132H^-induced tumorigenesis by degrading RBPJ [112].

The upregulation of Ribonucleotide reductase family member 2 (RRM2) expression is a common feature of many cancers, including GC [113]. Moreover, elevated levels of RRM2 are associated with poor prognosis [114,115,116]. APC/C^Cdh1^ targets RRM2 for ubiquitination and leads to its proteasomal degradation in the G1 phase to control its expression levels, while in the G2 phase, RRM2 expression is regulated by Cyclin F. The CY motif located at residues 49–51 and Thr33 in RRM2 are essential for binding to Cyclin F. Thr33 in RRM2 is phosphorylated by CDKs to promote its binding to Cyclin F [117,118]. RRM2 is ubiquitinated by SCF^Cyclin F^ and subsequently degraded by the proteasome in the G2 phase. Failure to degrade RRM2 leads to imbalanced dNTP pools, further promoting base mismatches during DNA synthesis, and facilitating cell mutations and tumor transformation (Table 2).

## 5. APC/C-Dependent Ubiquitination of Cyclins A and B in the M Phase of GC

On entry into mitosis, APC/C^CDC20^ begins to ubiquitinate Cyclin A, which is degraded during prometaphase while the spindle-assembly checkpoint (SAC) still protects Cyclin B [88,119]. Once all chromosomes are correctly attached, SAC silencing permits APC/C^CDC20^ to ubiquitinate Cyclin B and securin. Cyclin B degradation begins later, near metaphase, allowing anaphase to begin and mitotic exit to proceed [120,121,122,123]. APC/C-dependent ubiquitin-chain formation involves UBA1 and the E2 enzymes UBE2C and UBE2S. If APC/C^CDC20^ is inactive or Cyclin B1 is overexpressed, CRL2^ZYG11^ can provide an alternative pathway for Cyclin B1 ubiquitination [124].

Cyclin B1 is highly expressed in various cancers [125,126,127]. Zhang et al. demonstrated through Oncomine analysis that the mRNA expression levels of Cyclin B1 and Cyclin B2 are significantly higher in GC compared to normal tissues. Moreover, overexpression of Cyclin B1 mRNA has been confirmed in different subtypes of GC, suggesting that Cyclin B1 may contribute to guiding GC subtyping. The mRNA expression levels of Cyclin B1 are also significantly elevated in diffuse gastric adenocarcinoma and gastrointestinal adenocarcinoma [128]. Furthermore, as shown in a study of 482 GC patients by Begnami et al., patients with high Cyclin B1 expression had a poor five-year prognosis [125].

Cell cycle-related and expression-elevated protein in tumor (CREPT), also known as regulation of nuclear pre-mRNA domain-containing protein 1 (BRPRD1B), promotes GC cell proliferation and tumorigenesis by altering the cell cycle and is highly expressed in human GC. Ding et al. found that loss of CREPT leads to a significant accumulation of cells in the G2/M phase, prolonging the duration of the G2/M phase. Their further studies revealed that CREPT regulates the duration of the G2/M phase by modulating the transcription of Cyclin B1 in GC. CREPT can upregulate Cyclin B1 expression at the transcriptional level [129]. Meanwhile, Aurora B [130], a serine/threonine kinase, can phosphorylate CREPT at Ser145, and only phosphorylated CREPT at Ser145 can promote Cyclin B1 transcription [129]. Estrogen-related receptor alpha (ESRRA), also known as ERR1, ERRa, ERRalpha, ESRL1, and NR3B1, is an orphan nuclear receptor [131]. Giguère et al. found that ESRRA is a transcription factor that interacts with estrogen response elements (EREs) and estrogen-related response elements (ERREs), thereby controlling genes related to lipid and glucose metabolism and mitochondrial biogenesis [132]. Moreover, many studies have shown that ESRRA is associated with tumorigenesis, with overexpression of ESRRA being associated with immune infiltration in breast cancer, prostate cancer, and endometrial cancer [133,134,135,136]. Li et al. found that ESRRA is overexpressed in GC cells and promotes GC cells migration and proliferation by inducing epithelial–mesenchymal transition (EMT) and regulating Dosage suppressor of Nnf1 (DSN1) [137]. Li et al. further showed that ESRRA regulates the CDC25C/CDK1/Cyclin B1 pathway through DSN1 and that silencing ESRRA arrests GC cells at G2/M by reducing Cyclin B1 and CDK1 expression [137]. DSN1 is a core subunit of the conserved MIS12 kinetochore complex within the KMN network, which supports outer-kinetochore assembly and chromosome-spindle attachment during somatic mitosis [138].

**Figure 3:**
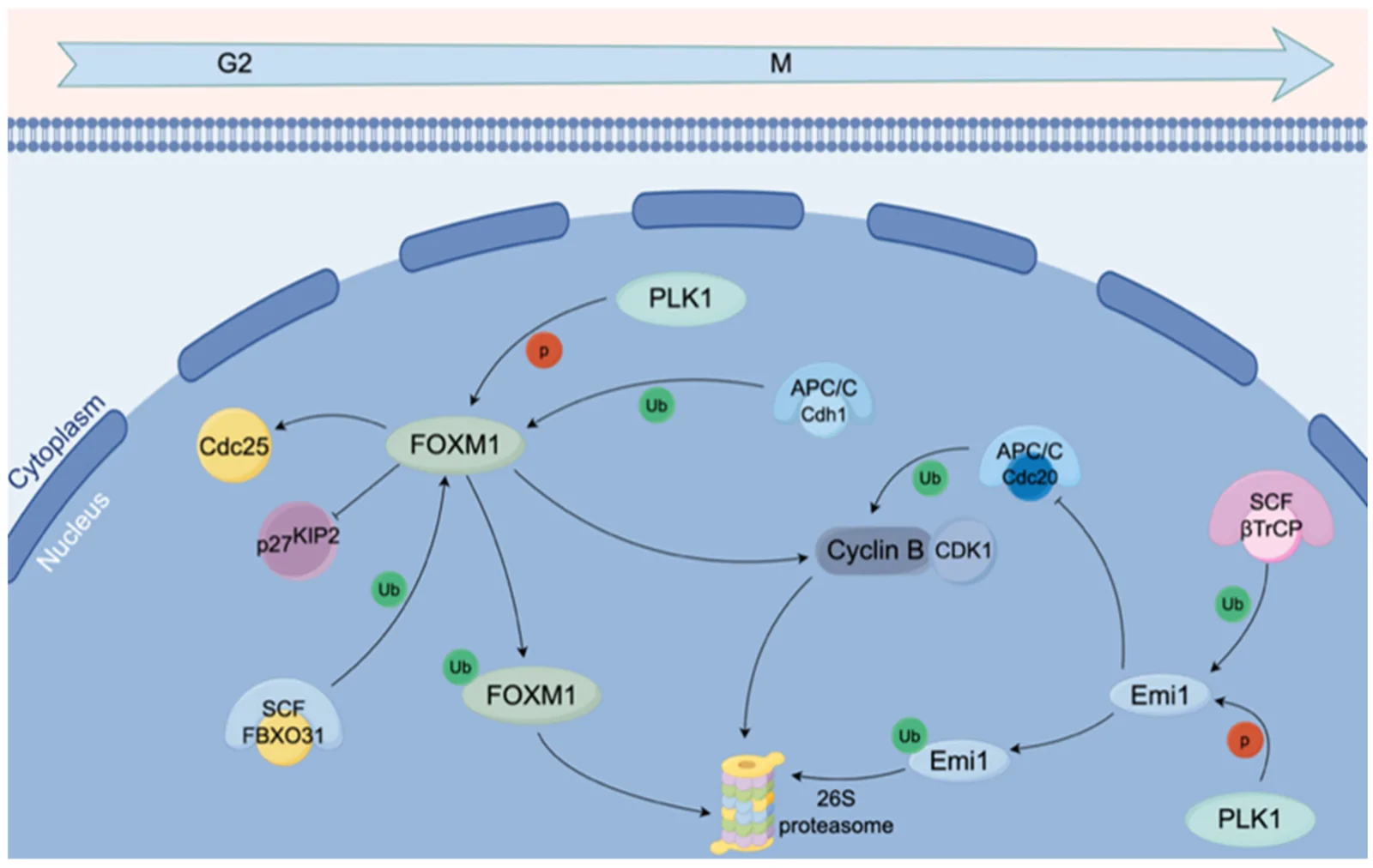
Regulation of FOXM1 and Cyclin B degradation. FOXM1 regulates CDK1, Cdc25, Cyclin B, and p27^KIP1^. SCF^FBXO31^ and APC/C^CDH1^ mediate FOXM1 degradation. PLK1 promotes SCF^βTrCP^-dependent degradation of Emi1, thereby relieving APC/C inhibition and allowing APC/C^CDC20^ to ubiquitinate Cyclin B for proteasomal degradation. Arrows indicate regulatory interactions and the direction of the pathway; T-bars indicate inhibition.

Forkhead box protein M1 (FOXM1) is a classical proliferation-related transcription factor, whose increased expression is observed in various human cancers, and elevated FOXM1 expression is associated with poor prognosis in most solid tumors. Okada et al. demonstrated high expression of FOXM1 in GC cells and its ability to regulate the expression of its downstream target genes CDK1, Cdc25, Cyclin B, and p27^KIP1^ to influence the GC cell cycle [139,140]. The degradation of FOXM1 involves the N-terminal KEN box (K-E-N-X-X-X-N) and destruction box (R-X-X-L-X-X-X-X-N) sequences, both of which can be recognized by the Ub–proteasome system. FOXM1 can regulate the transcription of UBE2C by binding to the promoter of the E2 ubiquitin-conjugating enzyme UBE2C. The upregulation of UBE2C transcription by FOXM1 has been observed in various tumors, including gastric adenocarcinoma, leading to its overexpression and contributing to abnormal cell cycle progression and tumorigenesis [141]. During the G2/M transition, E3 ubiquitin ligase SCF^FBXO31^ mediates the ubiquitination of FOXM1, leading to its degradation by the 26S proteasome, downregulating FOXM1 to maintain genome stability [142]. Upon exit from mitosis, FOXM1 degradation requires Cdh1, and APC/C^Cdh1^ interacts with FOXM1 and transfers Ubs carried by the E2 ubiquitin-conjugating enzymes UBE2C and UBE2S to FOXM1. This leads to the ubiquitination of FOXM1, making it recognizable for degradation by the proteasome, ensuring progression of the cell cycle [143]. Lin et al. discovered that treatment with 7-difluoromethoxyl-5,4′-di-n-octylgenistein (DFOG) led to significant accumulation of cells in the G2/M phase and a reduction in cells in the G1 phase in GC cell lines AGS and SGC-7901 [140]. DFOG downregulates FOXM1 expression levels in GC cells, while treatment with DFOG also leads to downregulation of FOXM1 target genes CDK1, Cdc25, and Cyclin B and upregulation of p27^KIP1^ expression. Monika et al. found that among the factors regulating mitosis, PLK1 may control the function of Survivin and FOXM1, which is critical for Aurora B activity and SAC signal transduction in cells treated with PLK1 inhibitors [144]. PLK1 inhibition downregulates Aurora B activity and Cyclin B degradation, which are key factors in mitotic slippage. Studies have shown that PLK1 overexpression predicts poor prognosis in GC [145]. During mitosis, PLK1 binds to Emi1 and phosphorylates Emi1, leading to its ubiquitination by SCF^βTrCP^; then Emi1 is degraded by the proteasome, thereby activating APC/C-mediated Cyclin B degradation (Figure 3) [146]. Additionally, clinical inhibitors of PLK1 such as BI2536 and BI6727 effectively inhibit cancer cell mitosis and induce apoptosis [144].

p21-activated kinase 1 (PAK1) is a serine/threonine protein kinase and one of the most studied effectors of the Rho family GTPases, Rac, cell division cycle 42 (Cdc42), and Cdc42 homologous protein (Chp) [147]. Elevated expression of PAK1 has been found in many human cancers, including GC, and studies have shown that PAK1 can regulate Cyclin B1 transcription through the NF-κB-dependent pathway, thereby modulating the cell cycle [148]. In GC cells, inhibition of PAK1 expression can suppress Cyclin B1 expression. Monika et al. found that overexpression of Cdc42 or Chp leads to a significant decrease in PAK1 protein levels in various cells. This decrease is mediated by the Ub–proteasome pathway [147]. And activation and autophosphorylation of PAK1 are essential for Chp-induced degradation, and mutations at phosphorylation sites T422A, S57A, and S198A/S203A significantly reduce the susceptibility of PAK1 to Chp-induced degradation (Table 3).

Besides the aforementioned E3 ubiquitin ligases, recent research has found that UBE2C can promote the Ub-mediated degradation of Cyclin B1 through multiple monoubiquitinations of Cyclin B1 [149,150]. In colorectal cancer, UBE2C can serve as a prognostic marker. Specific inhibition of UBE2C using bortezomib or siRNA can lead to the accumulation of Cyclin B1 and Cyclin A, causing cell cycle arrest at the G2/M phase [151]. UBE2S is another APC/C-associated E2 ubiquitin-conjugating enzyme that specifically extends Lys11 chains initiated by UBE2C. Its expression is upregulated in GC tissues, and high levels of UBE2S often indicate a poor prognosis for GC patients. Knockdown of UBE2S inhibits the growth of GC both in vivo and in vitro, and induces apoptosis in GC cells [152].

## 6. The Anti-Gastric Cancer Mechanism of Traditional Chinese Medicine Active Ingredients Interfering with Cyclin Ubiquitination Modification

The regulation of the active components of traditional Chinese medicine on cyclins can be carried out from two levels: one is to directly target the expression and function of cyclins; the second is to interfere with the ubiquitination degradation order of cyclins by interfering with multiple nodes of the ubiquitin–proteasome system (UPS), including E3 ubiquitin ligase, deubiquitinating enzyme and 26S proteasome. These two regulatory modes cooperate with each other to form a multi-level mechanism network for the active components of traditional Chinese medicine to inhibit the malignant progression of gastric cancer.

### 6.1. Regulation of Cyclin/CDK Expression and Cell Cycle Phenotypes Without Direct UPS Target Validation

#### 6.1.1. Downregulation of Cyclin D1 Expression Blocked the Initiation of G1/S Phase Transition

Cell cycle dysregulation is an important molecular basis for the occurrence and development of gastric cancer. Cyclin D/CDK4/6 complex is a key regulatory node that drives the transition from the G1 phase to the S phase. Under normal circumstances, Cyclin D1 binds to CDK4 or CDK6 to form an active complex and promotes the phosphorylation of retinoblastoma protein (Rb), and then releases the E2F transcription factor, initiates the transcription of DNA replication-related genes, and promotes cells to enter the S phase. A large number of studies have shown that Cyclin D1, CDK4 and CDK6 are generally highly expressed in gastric cancer tissues, and their abnormal activation is closely related to the unlimited proliferation, invasion and metastasis of tumor cells and poor prognosis [153]. In recent years, it has been found that a variety of natural active ingredients derived from traditional Chinese medicine can inhibit the phosphorylation of Rb protein by downregulating the expression of Cyclin D1, CDK4 and CDK6, thereby blocking the G1/S phase transition, and ultimately inhibiting the proliferation of gastric cancer cells and inducing apoptosis.

Dihydroartemisinin (DHA), an artemisinin derivative, is a representative compound of this type of action mechanism. Fan et al. found that DHA can significantly inhibit the proliferation and invasion of gastric cancer cells, and its mechanism is closely related to the inactivation of the Cyclin D1-CDK4-Rb signaling pathway. After DHA treatment, the expression of Cyclin D1 and CDK4 protein in gastric cancer cells was significantly decreased, and the phosphorylation level of Rb was inhibited, resulting in cell stagnation in the G0/G1 phase, thereby inducing apoptosis and growth inhibition [154]. Physalin B from Physalis also showed significant cell cycle regulation. Fang et al. found that Physalin B could significantly inhibit the proliferation of HGC-27 gastric cancer cells and induce G0/G1 phase arrest. Studies have shown that the compound plays an anti-tumor role by inhibiting the formation of the Cyclin D1/CDK4/6 complex and blocking the cell cycle initiation process [155]. Germacrone, a sesquiterpene component from Curcumae Rhizoma, can also target the key link of G1/S conversion. Wu et al. showed that the proliferation of gastric cancer cells was significantly inhibited after treatment with germacrone, and there was obvious G0/G1 phase accumulation. Mechanism studies have shown that it can downregulate the expression levels of Cyclin D1, CDK4 and CDK6, block cell cycle progression, and further induce mitochondrial-dependent apoptosis pathway activation [156]. The carotenoid component β-Cryptoxanthin has also been shown to have a similar effect. Gao et al. found that β-cryptoxanthin could significantly induce G0/G1 phase arrest in AGS and SGC-7901 gastric cancer cells, and reduce the expression levels of Cyclin D1, Cyclin E, CDK4 and CDK6 proteins. Studies suggest that its anti-proliferative effect is partly dependent on the negative regulation of the Cyclin D1/CDK4/6 axis after AMPK signal inactivation [157]. In recent years, the study of ginsenoside Rg3 in gastric cancer has been deepened. Recent studies have shown that 20(S)-ginsenoside Rg3 can downregulate the expression of Cyclin D1 and CDK4 by interfering with the transcriptional activity of E2F, and inhibit gastric cancer cells from the G1 phase to the S phase, thus exerting a significant anti-proliferative effect [158]. In addition, Lycium barbarum polysaccharide has also been found to inhibit the proliferation of MGC-803 cells, reduce the expression levels of Cyclin D and Cyclin E, and arrest cells in the G0/G1 phase [159].

In addition to the above compounds, although some plant extracts have not yet been found to clearly contain a single active ingredient, they also show obvious Cyclin D1/CDK4/6 regulatory effects. For example, the n-butanol extract of Ardisia villosa can induce G0/G1 phase arrest in gastric cancer cells and significantly downregulate the expression of cell cycle-related genes such as CDK4 and CDK6 [160].

Overall, Cyclin D1/CDK4/6 complex is the core driver of G1/S phase transition in gastric cancer cells. A large number of studies have confirmed that natural active ingredients derived from traditional Chinese medicine can inhibit Rb phosphorylation and E2F release by reducing the expression of Cyclin D1, CDK4 and CDK6, thereby blocking the cell cycle initiation process, and ultimately inhibiting proliferation, inducing apoptosis and enhancing the sensitivity of anti-tumor therapy. This mechanism not only reveals the important molecular basis of the active components of traditional Chinese medicine for interfering with the cell cycle of gastric cancer, but also provides a theoretical basis for the development of new CDK4/6-targeted drugs.

#### 6.1.2. Targeting Cyclin E/CDK2 and Cyclin A/CDK2: Inhibit S Phase Progression

The S phase of the cell cycle is a critical stage of DNA replication and chromosome synthesis, and its smooth progress depends on the sequential activation of Cyclin E/CDK2 and Cyclin A/CDK2 complexes. Cyclin E/CDK2 is mainly responsible for initiating G1/S phase transition and triggering DNA replication initiation, while Cyclin A/CDK2 maintains S phase DNA synthesis and replication fork stability. Studies have shown that Cyclin E and Cyclin A are often abnormally highly expressed in gastric cancer tissues. Their continuous activation can promote the unlimited proliferation of tumor cells and is closely related to tumor invasion, metastasis and poor prognosis [161,162,163]. At present, a variety of active ingredients derived from traditional Chinese medicine and natural medicine have been shown to induce S phase arrest by interfering with the Cyclin E/CDK2 and Cyclin A/CDK2 signaling axes, thereby inhibiting the proliferation of gastric cancer cells and promoting apoptosis.

Among them, Chinese propolis is a representative natural product with relatively complete evidence at present. Jiang et al. found that the ethanol extract of Chinese propolis could significantly inhibit the proliferation of gastric cancer cells and induce cell cycle arrest in the S phase. Further mechanism studies have shown that the expression of Cyclin E/CDK2 and Cyclin A/CDK2 complexes was significantly reduced after propolis treatment, and DNA replication-related proteins were inhibited, eventually leading to the failure of cells to complete DNA synthesis and enter the subsequent cycle stage, thereby inducing apoptosis [164]. This study directly demonstrated the anti-gastric cancer effect of natural products by regulating Cyclin E/CDK2 and Cyclin A/CDK2 to block the S phase process.

Celery seed extract (CSE) also showed significant S phase regulation. Gao et al. observed in BGC-823 gastric cancer cells that a large number of cells accumulated in the S phase after CSE treatment, accompanied by a significant increase in apoptosis. Western blot results showed that the expression levels of Cyclin A and CDK2 were significantly downregulated, suggesting that they interfere with DNA replication by inhibiting the activity of the Cyclin A/CDK2 complex, thereby inducing cell cycle arrest [165]. Since Cyclin A/CDK2 is the core regulatory factor that maintains the S phase process, this study provides an important basis for natural plant-derived components to target S phase regulation.

In addition, the ethanol extract of Cordyceps cicadae has also been shown to interfere with the S phase process. Xie et al. reported that the extract could significantly inhibit the proliferation of SGC-7901 gastric cancer cells and induce cell cycle arrest in the S phase. The study found that the expression of CDK2 decreased after treatment, and the expression of Cyclin A2 and Cyclin E changed significantly, which led to the blocking of DNA replication and further induced endoplasmic reticulum stress and apoptosis [166]. This result indicates that the active components of Chinese medicine fungi can also exert anti-gastric cancer effects through the Cyclin A/CDK2 and Cyclin E/CDK2 regulatory networks.

It is worth noting that although some natural products do not use S phase arrest as the main effect, they still involve Cyclin E/CDK2 and Cyclin A/CDK2 regulation. For example, cantharidin mainly induces G2/M phase arrest in gastric cancer cells, but researchers have found that it can affect the expression of Cyclin E/CDK2 and Cyclin A/CDK2 complexes, thereby interfering with the DNA replication process [167]. In addition, Geranium thunbergii extract can inhibit the activity of the CDK2/Cyclin E complex and block G1/S transition, which further verifies the potential application value of natural drugs targeting the Cyclin E/CDK2 axis [168].

In general, a variety of active ingredients from traditional Chinese medicine can inhibit the formation of related complexes and kinase activity by downregulating the expression of Cyclin E, Cyclin A and CDK2, resulting in blocked DNA replication and S phase arrest, and ultimately inducing apoptosis and inhibiting tumor proliferation. This mechanism not only enriches the theoretical basis of the anti-gastric cancer effect of the active ingredients of traditional Chinese medicine, but also suggests that Cyclin E/CDK2 and Cyclin A/CDK2 may become an important connection node for the follow-up study of ‘ubiquitination-dependent cyclin degradation’, which provides a research direction for further exploring the regulation of UPS-mediated cyclin homeostasis by traditional Chinese medicine.

#### 6.1.3. Targeting Cyclin B1/CDK1: Blocking G2/M Phase Transition

The G2/M phase is the key division preparation stage after DNA replication is completed in the cell cycle, which is precisely regulated by the Cyclin B1/CDK1 (also known as Cdc2) complex. Cyclin B1 binds to CDK1 to form a mature complex that drives cells from G2 phase to mitosis. In gastric cancer, Cyclin B1/CDK1 is usually highly expressed, and its abnormal activation not only promotes cell proliferation, but also is closely related to enhanced invasion and metastasis and poor prognosis.

In recent years, studies have found that a variety of traditional Chinese medicine and natural active ingredients can inhibit kinase activity by downregulating the expression of Cyclin B1 and CDK1, thereby blocking the G2/M phase transition, inhibiting the proliferation of gastric cancer cells and inducing apoptosis. Germacrone can significantly induce G2/M phase arrest in BGC-823 gastric cancer cells, while the expression levels of Cyclin B1 and CDK1 are decreased, accompanied by increased apoptosis [156]. Similarly, Osthole downregulated Cyclin B1 and CDK1 by inhibiting the PI3K/Akt pathway, induced G2/M phase arrest in gastric cancer cells, and significantly inhibited cell proliferation [169].

Honokiol also showed a potent G2/M blocking effect. In MGC-803 gastric cancer cells, after Honokiol treatment, the expression of Cyclin B1 and CDK1 protein decreased, the expression of p-CDC2 and p-cdc25 c was significantly upregulated, and the cells stagnated in the G2/M phase, accompanied by increased apoptosis [170]. By reducing the levels of Cyclin B1, Cyclin A2 and CDK1, 6-Gingerol not only induces G2/M phase arrest of HGC-27 cells, but also enhances the sensitivity of cells to radiotherapy, which provides an experimental basis for the combined application of active components of traditional Chinese medicine and radiotherapy [171].

Iridin induces G2/M phase arrest in AGS gastric cancer cells, downregulates the expression of Cyclin B1, CDK1 and Cdc25 C, and inhibits the preparation of DNA division [172]. Piperlongumine also exhibits the effect of inducing G2/M phase arrest and apoptosis by destroying the Cyclin B1/CDK1 axis in vitro and in vivo [173]. In addition, Cantharidin can also induce G2/M phase arrest in SGC-7901 and BGC-823 gastric cancer cells by regulating the Cyclin B1/CDK1 complex [167]. Tanshinone IIA also induces G2/M phase arrest in AGS cells by inhibiting CDK1/Cyclin B1 expression and activating the p38 and JNK pathways [174].

### 6.2. Regulating E3 Ubiquitin-Ligase Activity: Reconstructing the Degradation Order of Cyclins

The expression of cyclins is significantly time-dependent, and their synthesis and degradation are strictly controlled by the ubiquitin–proteasome system (UPS). In normal cells, cyclins are recognized and labeled by E3 ubiquitin ligase after completing specific cell cycle functions, and then degraded by 26S proteasome to ensure orderly cell cycle progression. However, during the development of gastric cancer, a variety of E3 ubiquitin ligases are abnormally expressed or dysfunctional, resulting in abnormal accumulation of key cyclins such as Cyclin D1, Cyclin E and Cyclin B1, promoting cell proliferation and enhancing the malignant phenotype [48,175].

In recent years, studies have shown that active components of traditional Chinese medicine can not only directly inhibit the expression of cyclins, but also reconstruct cyclin homeostasis by regulating the activity of E3 ubiquitin ligase, thereby restoring the cell cycle regulatory network. Depending on the mechanism of action, these E3 ligases mainly include the Skp2-SCF complex, which promotes the degradation of CDK inhibitors, and FBXW7, which mediates Cyclin E degradation.

#### 6.2.1. Inhibition of Skp2-SCF Complex: Restoration of Endogenous CDK Inhibitors

Skp2 is the core F-box protein of the SCF (Skp1-Cullin-F-box) complex, and is highly expressed in gastric cancer. It can target the CDK inhibitor p27^KIP1^ for ubiquitination degradation and drive gastric cancer cell proliferation [176]. After Skp2 is inhibited, it induces cell cycle arrest by upregulating p27, p21, p57 and downregulating Cyclin E and CDK2 [177]. Therefore, it has become an important strategy for the development of anti-gastric cancer drugs for interfering with the activity of the Skp2-SCF complex and restoring the expression levels of endogenous CDK inhibitors such as p27/p21.

Rhus verniciflua Stokes ethanol extract has been investigated for its effects on cell cycle regulation in gastric cancer cells. Kim et al. reported that the extract inhibited cell proliferation and induced G1 phase arrest, accompanied by increased p27KIP1 expression [178]. However, the study did not directly examine SKP2 activity, p27 ubiquitination, or the interaction between the extract and the SCFSKP2 complex. The observed p27 accumulation is therefore consistent with reduced SKP2–p27 pathway activity but does not establish SKP2 inhibition as the underlying mechanism. Similarly, pine needle hexane extract induced cell cycle arrest and cellular senescence together with increased p27 expression in gastric cancer cells [179]. Although these findings suggest possible involvement of the SKP2–p27 axis, the contribution of SKP2 was not tested directly.

Taken together, these studies indicate that preservation of endogenous CDK inhibitors may contribute to the cell cycle effects of these natural extracts. In contrast to approaches that primarily alter the expression of individual cyclins, modulation of the SCFSKP2–p27 axis could provide an upstream means of restoring negative cell cycle control. Nevertheless, the available evidence remains indirect and is largely limited to gastric cancer cell-line experiments. Direct measurements of p27 ubiquitination, SKP2-dependent rescue experiments, target-engagement assays, and in vivo validation will be required before SCFSKP2 can be regarded as a confirmed molecular target of these extracts.

#### 6.2.2. Regulation of FBXW7: Validated and Proposed Mechanisms

FBXW7 (F-box and WD repeat domain-containing 7) is a tumor suppressor SCF E3 ubiquitin ligase that specifically recognizes and degrades a variety of oncogenic proteins, including Cyclin E and c-Myc. In gastric cancer, FBXW7 functional inactivation or downregulation leads to the continuous accumulation of Cyclin E and c-Myc, which promotes S phase overactivation, DNA replication pressure and genomic instability, and ultimately accelerates tumor proliferation and malignant progression [180,181].

Among the compounds discussed in this review, demethylzeylasteral provides the clearest evidence of FBXW7’s involvement in gastric cancer. Treatment increased FBXW7 expression, enhanced the ubiquitination and proteasomal degradation of c-Myc, and suppressed tumor growth; these effects were weakened following FBXW7 depletion [182]. This study therefore supports an FBXW7-dependent mechanism, although the substrate examined was c-Myc rather than Cyclin E. By comparison, curcumin modulated the miR-21/PTEN/Akt pathway in MGC-803 cells [183], while berberine combined with low-glucose treatment activated PP2A/GSK3β signaling and reduced MCL-1 expression in gastric cancer cells and xenograft models [184]. In a separate colorectal cancer model, berberine upregulated PTEN and inhibited Notch1/PI3K/Akt/mTOR signaling, resulting in G1-phase arrest in SW480 cells [185]. None of these studies directly examined FBXW7 activity, FBXW7-dependent Cyclin E ubiquitination, or Cyclin E turnover. Accordingly, any proposed association between these interventions and the FBXW7–Cyclin E pathway remains indirect and requires experimental verification.

Several non-coding RNAs have nevertheless been linked to the regulation of F-box ligases in gastric cancer. Reported examples include the miR-17/20a–FBXO31, miR-25–FBXW7, and hsa_circ_001988/miR-197-3p–FBXW7 pathways [31,39,77], as well as the HNF1A-AS1/miR-661/CDC34 and circNFATC3/TRIM25 regulatory axes [54,72]. These findings establish a potential connection between non-coding RNA signaling and the ubiquitin–proteasome system. However, none of the compounds reviewed here have been demonstrated to regulate cyclin turnover through a complete ncRNA–E3 ligase–cyclin pathway. Although curcumin altered miR-21/PTEN/Akt signaling, the corresponding study did not examine E3-ligase activity or cyclin ubiquitination [183]. Whether TCM-derived compounds influence cyclin stability through ncRNA-dependent regulation of E3 ligases therefore remains an open question.

### 6.3. Direct Inhibition of 26S/20S Proteasome Activity

The ubiquitin–proteasome system (UPS) is the main pathway for regulated intracellular protein degradation [186]. The 26S proteasome comprises a 20S catalytic core and 19S regulatory particles and exhibits chymotrypsin-like, trypsin-like, and caspase-like activities [187]. In the studies reviewed below, reduced cellular 20S or 26S activity was considered direct functional evidence of proteasome inhibition. Such assays do not, however, demonstrate binding to the purified proteasome or target engagement in vivo [188,189,190].

Huachansu is a Chinese patent medicine prepared from Chinese toad skin. Deng et al. prepared compound-composed Chinese medicine of Huachansu (CCMH) from eight Huachansu-derived bufadienolides: arenobufagin, bufalin, bufotalin, cinobufagin, cinobufotalin, gamabufotalin, resibufogenin, and telocinobufagin. In GC cells, CCMH reduced cellular 20S proteasome activity and induced ROS-associated G2/M arrest and apoptosis [188]. Although the study directly measured cellular proteasome activity, it did not assess binding to the purified proteasome or target engagement in vivo.

Gambogic acid is a xanthone obtained from the dried resin of *Garcinia hanburyi* (Tenghuang). Direct proteasome inhibition, including CYP2E1-dependent metabolic activation, was demonstrated in non-GC models [189]. In MGC-803 and SNU-16 GC cells, however, the cited studies principally reported apoptosis and autophagy [191,192]. CHIP-mediated ABCB1 degradation and reversal of multidrug resistance were also demonstrated in other epithelial cancer models [193]. Direct proteasome inhibition was therefore shown outside GC, whereas the GC studies provided phenotypic evidence.

**Figure 4:**
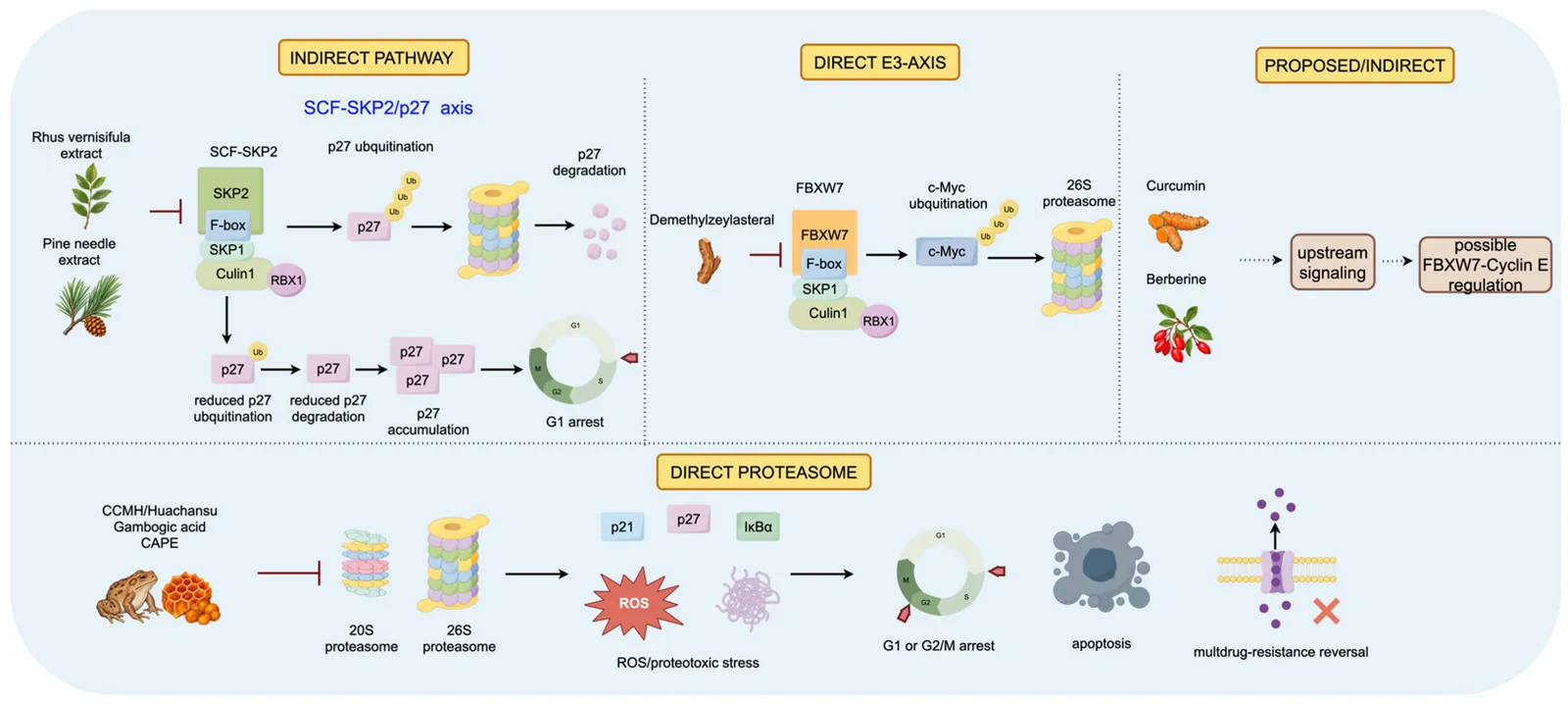
Effects of TCM-derived and natural products on UPS-related pathways in GC, classified by the level of mechanistic evidence. Solid lines represent mechanisms supported by ubiquitination or proteasome-activity assays; dotted lines represent indirect or proposed mechanisms. Demethylzeylasteral directly promotes FBXW7-dependent c-Myc ubiquitination in GC. The proposed links of curcumin and berberine to the FBXW7-Cyclin E pathway are based on upstream signaling data. CCMH and CAPE reduce cellular proteasome activity in GC cells. Direct proteasome evidence for gambogic acid was obtained outside GC, whereas the GC studies mainly reported phenotypic effects. Arrows indicate regulatory interactions and the direction of the pathway; T-bars indicate inhibition.

Caffeic acid phenethyl ester (CAPE), a major component of propolis, reduced 26S proteasome activity in parental and drug-resistant MKN45 cells, with the greatest reduction in chymotrypsin-like activity. In doxorubicin-resistant cells, CAPE also reduced PSMB1-PSMB5 and PSMB8-PSMB10 mRNA levels and increased sensitivity to doxorubicin and cisplatin [190] (Figure 4, Table 4). These cellular assays directly support proteasome inhibition, but the study was limited to in vitro experiments and included neither a binding assay nor an in vivo target-engagement analysis.

The strength of the evidence varies across Section 6. Demethylzeylasteral is supported by direct evidence for regulation of the FBXW7-c-Myc pathway in GC [182], and CCMH and CAPE directly reduced cellular proteasome activity [188,193]. Most compounds discussed in Section 6.1 were assessed only through changes in cyclin/CDK expression and cell cycle distribution. The proposed links of curcumin and berberine to FBXW7-Cyclin E have likewise not been verified directly [154,155,156,157,158,159,160,161,162,163,164,165,166,167,168,169,170,171,172,173,174,175,176,177,178,179,180,181,182,183,184,185,186,187,188,189,190,191,192,193]. These preclinical findings do not establish clinical efficacy or superiority over CDK-targeted therapy.

## 7. Conclusions

Ubiquitination serves as a fundamental post-translational mechanism that orchestrates the precise spatiotemporal degradation of cyclins, thereby ensuring orderly cell cycle progression. In gastric cancer, the frequent aberration of E3 ubiquitin ligases—such as FBXW7, FBXO4, SKP2, AMBRA1, and TRIM family members—and the involvement of regulatory non-coding RNAs (e.g., miR-17/20a, miR-25, hsa_circ_001988, HNF1A-AS1) disrupt the ubiquitination and proteasomal turnover of Cyclins D, E, A, and B, leading to their sustained overexpression. This dysregulation promotes GC cell proliferation, invasion, metastasis, and chemoresistance, ultimately correlating with poor patient prognosis.

Our synthesis reveals that each cell cycle phase is governed by specific ubiquitin-ligase–cyclin pairs: the G1 phase (SCF-mediated degradation of Cyclin D/E), S phase (Cyclin A regulation via SKP2 and other ligases), G2 phase (Cyclin F-SCF and Cyclin A control), and M phase (APC/C-dependent Cyclin B destruction). These insights not only deepen our understanding of GC pathogenesis but also identify multiple potential intervention points.

Among the TCM-derived and natural products reviewed, direct mechanistic support in GC is limited to a small subset. Demethylzeylasteral promotes FBXW7-dependent c-Myc ubiquitination [182], whereas CCMH and CAPE reduce cellular proteasome activity [188,193]. The remaining studies largely reported changes in cyclin/CDK expression or E3-ligase-associated pathways and provided phenotypic or indirect evidence [154,155,156,157,158,159,160,161,162,163,164,165,166,167,168,169,170,171,172,173,174,175,176,177,178,179,180,181,182,183,184,185,186,187,188,189,190,191,192,193]. Proposed mechanisms require direct testing before these compounds can be regarded as UPS-targeted therapies.

Most reports examined a single GC cell line, and experiments performed in different laboratories varied in dose, treatment duration, and endpoints [154,155,156,157,158,159,160,161,162,163,164,165,166,167,168,169,170,171,172,173,174,175,176,177,178,179,180,181,182,183,184,185,186,187,188,189,190,191,192,193]. The magnitude of the reported effects therefore cannot be compared directly. In vivo validation was available for only a subset, and many studies did not assess compound-target binding, on-target ubiquitination, pharmacodynamic exposure, dose–response relationships, or systematic toxicity.

GC is molecularly heterogeneous. TCGA classified GC into EBV-positive, microsatellite instability, genomically stable, and chromosomal instability subtypes. ACRG identified subtypes with distinct molecular features, recurrence patterns, and clinical outcomes [194,195]. Future studies should first examine authenticated panels of GC cell lines representing the major molecular and histological subtypes, then validate the findings in organoid and in vivo models.

Poor oral exposure and rapid metabolism further limit the clinical use of several natural compounds. After healthy volunteers received 10–12 g oral curcumin, free curcumin was rarely detected, and conjugated metabolites predominated [196]. In rats, berberine underwent extensive intestinal first-pass elimination and had an absolute oral bioavailability of 0.36% [197]; the corresponding value for gambogic acid was 0.25–0.32% [198]. These pharmacokinetic data indicate that the micromolar concentrations used in cultured GC cells may not correspond to concentrations achievable in tumors.

Future studies should combine direct target-engagement assays and rescue experiments with standardized comparisons across GC models and appropriate normal gastric cell controls. They should also incorporate pharmacokinetic/pharmacodynamic measurements, formulation studies, dose–response and toxicity analyses, and in vivo validation before translational or clinical conclusions are drawn [194,195,196,197,198].

## Figures and Tables

**Table 1:** E2 ubiquitin-conjugating enzyme and E3 ubiquitin ligase in G1 phase.

Cyclin Context/Direct Substrate	E3 Ubiquitin-Ligase Complex (Substrate Receptor or Co-Activator)	E2 Enzyme	Relationship to Cyclin Regulation	References
Cyclin D1 (direct substrate)	SCF^FBXO4^-αB-crystallin	UBE2D family	Direct ubiquitination of phosphorylated Cyclin D1 and 26S proteasomal degradation	[27,29]
Cyclin D1 (direct substrate)	SCF^FBXO31^	UBE2D family	Direct Cyclin D1 ubiquitination; miR-17/20a suppress FBXO31 in GC	[30,31]
Cyclins D1-D3 (direct substrates)	CRL4^AMBRA1^	UBE2D1; UBE2G1; UBE2G2	Direct ubiquitination and degradation of D-type cyclins	[32,33]
Cyclin D1 (direct substrate)	MG53/TRIM72	UBE2D; UBE2N	Direct K48-linked Cyclin D1 ubiquitination	[38]
Cyclin D1 context (indirect)	SCF^FBXW7^	Not established in cited GC study	hsa_circ_001988/miR-197-3p regulates FBXW7	[39]
Cyclin D1 context (indirect via CKIs)	SCF^SKP2^	CDC34/UBE2R1	Ubiquitination of p21/p27 and other CKIs indirectly stabilizes Cyclin D-CDK activity	[47,48,49]
Cyclin D1 context (indirect via p53)	TRIM59	Not specified	p53 ubiquitination alters downstream p21/Cyclin D1; Cyclin D1 is not the direct substrate	[50]
Cyclin D1 context (indirect via β-catenin)	TRIM58	Not specified	β-catenin ubiquitination reduces Cyclin D1 transcription; UBAP2L is not an E2 enzyme	[52,53]
Cyclin D1 context (indirect via IGF2BP3)	TRIM25	Not specified	circNFATC3 limits TRIM25-mediated IGF2BP3 ubiquitination, thereby stabilizing Cyclin D1 mRNA	[54]
Cyclin D1 context (indirect via ERK/MAPK)	RNF181	Not specified	ERK/MAPK and Cyclin D1-CDK4 activity are affected	[55]
Cyclin E (direct substrate)	SCF^FBXW7^	CDC34/UBE2R1	Direct phosphorylation-dependent Cyclin E ubiquitination and degradation	[56,63,64,65]

**Table 2:** E2 ubiquitin-conjugating enzyme and E3 ubiquitin ligase in G2 phase.

Cyclin Context/Direct Substrate	E3 Ubiquitin-Ligase Complex (Substrate Receptor or Co-Activator)	E2 Enzyme	Relationship to Cyclin Regulation	References
Cyclin A (direct substrate)	APC/C^CDH1^	UBE2C; UBE2S	Direct Cyclin A ubiquitination opposed by USP37 during G1/S	[80,81]
Cyclin A context (indirect via p27/CKIs)	SCF^SKP2^	CDC34/UBE2R1; UBE2D	CKI ubiquitination activates Cyclin A-CDK2	[82,83,86]
Cyclin A (transcriptional regulation)	RNF6	Not applicable to transcriptional mechanism	RNF6 upregulates CCNA1/CREBBP transcription; not direct Cyclin A ubiquitination	[98]
Cyclin A/B context via YBX1	NEDD4L	Not specified in cited study	YBX1 ubiquitination indirectly affects cyclin transcription; evidence is from a cardiac, not a GC, model	[99,100]
E2F1 (non-cyclin substrate)	SCF^Cyclin F^	CDC34/UBE2R1	Direct non-cyclin substrate ubiquitination; evidence is not GC-specific	[106]
E2F7/E2F8 (non-cyclin substrates)	SCF^Cyclin F^	Not specified in cited studies	Direct non-cyclin substrate ubiquitination; evidence is not GC-specific	[107,108]
RBPJ (non-cyclin substrate)	SCF^Cyclin F^	Not specified in cited study	Direct RBPJ ubiquitination indirectly limits IDH1-related tumorigenesis	[112]
RRM2 (non-cyclin substrate)	SCF^Cyclin F^	Not specified in cited study	Direct RRM2 ubiquitination regulates dNTP pools; evidence is not GC-specific	[117,118]

**Table 3:** Direct and indirect ubiquitination-related mechanisms during the M phase.

Cyclin Context/Direct Substrate	E3 Ubiquitin-Ligase Complex (Substrate Receptor or Co-Activator)	E2 Enzyme	Relationship to Cyclin Regulation	References
Cyclin A (direct substrate; prometaphase)	APC/C^CDC20^	UBE2C; UBE2S	Direct ubiquitination; Cyclin A is degraded before Cyclin B	[88,119]
Cyclin B (direct substrate)	APC/C^CDC20^	UBE2C; UBE2S	Direct ubiquitination after the spindle-assembly checkpoint is satisfied	[120,121,122,123]
Cyclin B1 (alternative direct pathway)	CRL2^ZYG11^	Not specified	Direct Cyclin B1 ubiquitination when APC/C is inactive or Cyclin B1 is overexpressed	[124]
Cyclin B context; FOXM1 substrate	SCF^FBXO31^	Not specified	Indirect cyclin regulation through FOXM1 ubiquitination and degradation	[142]
Cyclin B context; FOXM1 substrate	APC/C^CDH1^	UBE2C; UBE2S	Indirect cyclin regulation through FOXM1 turnover at mitotic exit	[143]
APC/C activation; Emi1 substrate	SCF^βTrCP^	Not specified	PLK1-dependent Emi1 degradation relieves APC/C inhibition	[146]

**Table 4:** All peer-reviewed TCM-derived and natural agents or formulations discussed in Section 6, including their sources, cyclin/UPS relationships, evidence categories, experimental models, and main findings.

Agent/Formulation and TCM or Natural Source	Cyclin/UPS Node	Level of Mechanistic Evidence	Experimental Model and Main Finding	References
Dihydroartemisinin (DHA)—Qinghao (*Artemisia annua* derivative)	Cyclin D1-CDK4-Rb	Phenotypic evidence: reduced pathway proteins; ubiquitination was not examined	GC cell lines; in vitro G0/G1 arrest and apoptosis	[154]
Physalin B—Kuzhi (*Physalis* source)	Cyclin D1-CDK4/6	Phenotypic evidence: changes in complex abundance/function; UPS target engagement was not examined	HGC-27 cells; in vitro G0/G1 arrest	[155]
Germacrone—Ezhu (Curcumae Rhizoma)	Cyclin D1-CDK4/6 and Cyclin B1-CDK1	Phenotypic evidence: changes in cyclin/CDK expression; ubiquitination was not examined	GC cells; in vitro cell cycle arrest and apoptosis	[156]
β-Cryptoxanthin—dietary carotenoid; no specific TCM source in the cited GC study	Cyclins D1/E; CDK4/6	Phenotypic evidence: protein changes and AMPK-associated arrest	AGS and SGC-7901 cells; in vitro	[157]
20(S)-Ginsenoside Rg3—Renshen/Hongshen (*Panax ginseng*)	E2F; Cyclin D1-CDK4	Phenotypic and transcriptional evidence; cyclin ubiquitination was not examined	GC cells; E2F-DP-dependent antiproliferative effect	[158]
*Lycium barbarum* polysaccharide—Gouqizi	Cyclins D/E	Phenotypic evidence: reduced cyclin expression; UPS activity was not examined	MGC-803 cells; in vitro G0/G1 arrest	[159]
*Ardisia villosa* n-butanol extract—Xuexiahong	CDK4/6; G1 arrest	Phenotypic evidence: changes in cell cycle genes and senescence	GC cells; in vitro	[160]
Chinese propolis ethanol extract—Fengjiao	Cyclin E/A-CDK2	Phenotypic evidence: reduced complex proteins; ubiquitination was not examined	GC cells; in vitro S phase arrest	[164]
Celery seed extract—Qincai zi	Cyclin A-CDK2	Phenotypic evidence: reduced protein expression; UPS activity was not examined	BGC-823 cells; in vitro S phase arrest	[165]
*Cordyceps cicadae* ethanol extract—Chanhua	Cyclins A2/E; CDK2	Phenotypic evidence: changes in protein expression; UPS activity was not examined	SGC-7901 cells; in vitro S phase arrest	[166]
Cantharidin—Banmao (Mylabris-derived)	Cyclins A/E/B1; CDK1/2	Phenotypic evidence: changes in cyclin/CDK expression; ubiquitination was not examined	SGC-7901 and BGC-823 cells; in vitro G2/M arrest	[167]
*Geranium thunbergii* extract—Laoguancao	Cyclin E-CDK2	Phenotypic evidence: regulation of G1/S signaling; UPS activity was not examined	GC cells; in vitro G1 arrest and apoptosis	[168]
Osthole—Shechuangzi (*Cnidium monnieri*)	Cyclin B1-CDK1; PI3K/Akt	Phenotypic and indirect signaling evidence; ubiquitination was not examined	GC cells; in vitro G2/M arrest	[169]
Honokiol—Houpo (Magnolia bark)	Cyclin B1-CDK1; Cdc25C	Phenotypic evidence: changes in protein expression and phosphorylation	MGC-803 cells; in vitro G2/M arrest	[170]
6-Gingerol—Shengjiang/Ganjiang (*Zingiber officinale*)	Cyclins B1/A2; CDK1	Phenotypic evidence: changes in cyclin/CDK expression; UPS activity was not examined	HGC-27 cells; in vitro radiosensitization	[171]
Iridin—Shegan (*Iris domestica*)	Cyclin B1-CDK1-Cdc25C	Phenotypic and indirect PI3K-Akt evidence; ubiquitination was not examined	AGS cells; in vitro G2/M arrest	[172]
Piperlongumine—Bibo (*Piper longum*)	Cyclin B1-CDK1	Phenotypic evidence: changes in pathway proteins and cell cycle distribution	GC cells plus xenograft; G2/M arrest and apoptosis	[173]
Tanshinone IIA—Danshen (*Salvia miltiorrhiza*)	Cyclin B1-CDK1; p38/JNK	Phenotypic and indirect signaling evidence; ubiquitination was not examined	AGS cells; in vitro G2/M arrest	[174]
*Rhus verniciflua* Stokes ethanol extract—Qishu	SCF^SKP2^-p27	Indirect UPS-associated evidence: p27 accumulation; target engagement and p27 ubiquitination were not examined	AGS cells only; G1 arrest and apoptosis	[178]
Pine needle hexane extract—Songzhen (*Pinus densiflora*)	SKP2-p27	Indirect/proposed mechanism: increased p27 expression; the role of SKP2 was not directly tested	GC cells only; arrest, senescence, and drug combination	[179]
Demethylzeylasteral—Leigongteng (*Tripterygium wilfordii*)	FBXW7-c-Myc	Direct E3-ligase evidence in GC: c-Myc ubiquitination and proteasomal degradation with FBXW7 dependence	GC cells plus xenograft; rescue/knockdown evidence	[182]
Curcumin—Jianghuang (purified curcumin)	miR-21/PTEN/Akt; proposed FBXW7-Cyclin E	Proposed mechanism based on upstream signaling; FBXW7 regulation and Cyclin E ubiquitination were not examined	MGC-803 cells; in vitro	[183]
Berberine—Huanglian/Huangbai and related sources (purified alkaloid)	PP2A/GSK3β/MCL-1; proposed FBXW7-Cyclin E	Indirect/proposed mechanism based on upstream signaling; FBXW7 regulation and Cyclin E turnover were not examined	MGC-803 cells plus xenograft under low-glucose conditions	[184]
CCMH—Huachansu (toad-skin-derived eight-component formulation)	20S proteasome	Direct evidence of cellular proteasome inhibition; purified-proteasome binding and in vivo target engagement were not examined	GC cells; 20S activity assay, ROS, G2/M arrest, apoptosis	[188]
Gambogic acid—Tenghuang (*Garcinia hanburyi* resin)	20S proteasome; CHIP-ABCB1	Direct proteasome inhibition in non-GC models; phenotypic evidence in GC	Non-GC direct mechanism; GC cell apoptosis/autophagy; MDR models	[189,191,192,193]
CAPE—Fengjiao (propolis constituent)	26S proteasome	Direct evidence of cellular proteasome inhibition; binding and in vivo target engagement were not examined	Parental and resistant MKN45 cells; in vitro chemosensitization	[190]

## Data Availability

No new data were created or analyzed in this study. Data sharing is not applicable to this article.
